# Membrane Insertion for the Detection of Lipopolysaccharides: Exploring the Dynamics of Amphiphile-in-Lipid Assays

**DOI:** 10.1371/journal.pone.0156295

**Published:** 2016-05-26

**Authors:** Loreen R. Stromberg, Nicolas W. Hengartner, Kirstie L. Swingle, Rodney A. Moxley, Steven W. Graves, Gabriel A. Montaño, Harshini Mukundan

**Affiliations:** 1 Center for Biomedical Engineering, University of New Mexico, Albuquerque, New Mexico, United States of America; 2 Physical Chemistry and Applied Spectroscopy, Los Alamos National Laboratory, Los Alamos, New Mexico, United States of America; 3 The New Mexico Consortium, Los Alamos, New Mexico, United States of America; 4 Theoretical Biology and Biophysics, Los Alamos National Laboratory, Los Alamos, New Mexico, United States of America; 5 Center for Integrated Nanotechnologies, Los Alamos National Laboratory, Los Alamos, New Mexico, United States of America; 6 School of Veterinary Medicine and Biomedical Sciences, University of Nebraska-Lincoln, Lincoln, Nebraska, United States of America; Consejo Superior de Investigaciones Cientificas, SPAIN

## Abstract

Shiga toxin-producing *Escherichia coli* is an important cause of foodborne illness, with cases attributable to beef, fresh produce and other sources. Many serotypes of the pathogen cause disease, and differentiating one serotype from another requires specific identification of the O antigen located on the lipopolysaccharide (LPS) molecule. The amphiphilic structure of LPS poses a challenge when using classical detection methods, which do not take into account its lipoglycan biochemistry. Typically, detection of LPS requires heat or chemical treatment of samples and relies on bioactivity assays for the conserved lipid A portion of the molecule. Our goal was to develop assays to facilitate the direct and discriminative detection of the entire LPS molecule and its O antigen in complex matrices using minimal sample processing. To perform serogroup identification of LPS, we used a method called membrane insertion on a waveguide biosensor, and tested three serogroups of LPS. The membrane insertion technique allows for the hydrophobic association of LPS with a lipid bilayer, where the exposed O antigen can be targeted for specific detection. Samples of beef lysate were spiked with LPS to perform O antigen specific detection of LPS from *E*. *coli* O157. To validate assay performance, we evaluated the biophysical interactions of LPS with lipid bilayers both in- and outside of a flow cell using fluorescence microscopy and fluorescently doped lipids. Our results indicate that membrane insertion allows for the qualitative and reliable identification of amphiphilic LPS in complex samples like beef homogenates. We also demonstrated that LPS-induced hole formation does not occur under the conditions of the membrane insertion assays. Together, these findings describe for the first time the serogroup-specific detection of amphiphilic LPS in complex samples using a membrane insertion assay, and highlight the importance of LPS molecular conformations in detection architectures.

## Introduction

Shiga toxin-producing *Escherichia coli* (STEC) is an important cause of foodborne illness with cases attributable to beef and fresh produce, among other sources [1]. There are many serotypes of STEC with a wide range of virulence, which are capable of infecting humans. Identification in part has relied upon detection of serotype, which in turn, relies on the identification of external biomarkers on the bacterial cell.

Lipopolysaccharide (LPS) is the primary component of the outer membrane of Gram-negative bacteria, and a key stimulator of the mammalian innate immune system [2–5]. LPS is among a class of molecules called pathogen-associated molecular patterns (PAMPs). PAMPs are bacterial products, often with redundant molecular structure, that are recognized by many host immune receptors, e.g., Toll-like receptors [6]. The bacterial membrane of an *Escherichia coli* (*E*. *coli*) cell is comprised of approximately 10^6^ LPS molecules, or about 75% of the outer membrane [7–9]. LPS, and more specifically the lipid A moiety is also known as endotoxin, and can induce septic shock in a variety of mammalian hosts through the activation of monocytes and macrophages that release a series of inflammatory cytokines [10–15] in response to invading pathogens.

**Fig 1:**
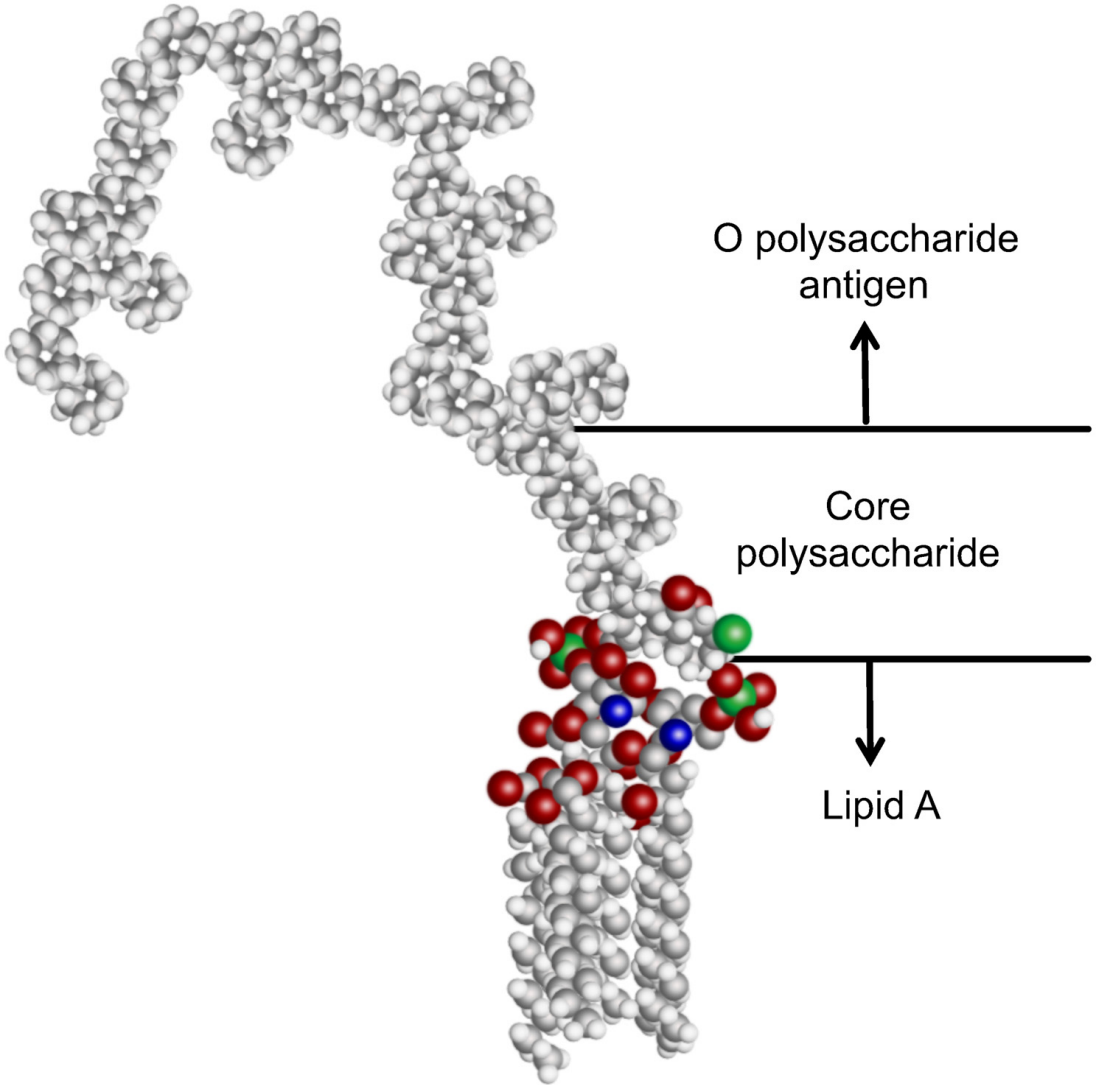
Representative structure of the molecular components of LPS. The conserved, hydrophobic lipid A group, core polysaccharide, and hypervariable O polysaccharide antigen. The lipid A group of most *E*. *coli* strains has 6 fatty acid tails which anchors LPS into the bacterial cell membrane, and is recognized by host receptor proteins.

The structure and signaling mechanism of LPS has been well studied [16]. LPS is a negatively charged amphiphilic molecule that consists of three primary components (Fig 1). The hydrophobic lipid A tail is a highly conserved molecule consisting of 6–7 fatty acid tails [8]. The endotoxic effects of lipid A [9,16,17] are initiated by the binding of this component to host receptors and serum binding proteins *in vivo* [13,18,19]. Lipid A is covalently attached to the less conserved core polysaccharide region, which in turn extends to the hypervariable O polysaccharide antigen (O-ag) [16,17,20,21]. Typically, the O-ag consists of 1–50 subunits made of 1–7 glycosyl residues [21,22]. Among different serotypes and species, the O-ag can vary greatly in both identity and degree of branching of the glycosyl residues [21]. This variability is therefore used for classifying a bacterial serotype. Interestingly, many of the PAMPs that stimulate host innate immune recognition, such as lipoarabinomannan from *Mycobacterium tuberculosis*, share a similar amphipathic structure [23,24]. Beyond LPS, detection of such amphiphilic signatures is critical to the understanding of host-pathogen biology.

Detection of LPS and identification of the O-ag is not always straightforward because of the variability in structure, and the possibility for conserved epitopes to present on multiple serogroups of LPS [16,21,25]. Detection methods for LPS typically focus on quantification of lipid A or its biological activity, rather than identification of serogroup [26,27]. While this method of detection is valuable for determining endotoxin contamination in sterile injectables and implantable devices, it provides little value for diagnostic applications. Immunoassays that are capable of antigen discrimination are optimized for detection of protein antigens and do not take into account the amphiphilic biochemistry of lipoglycans, causing low sensitivity and repeatability [28,29]. Factors such as conserved hydrophobic regions, micelle aggregation, poor binding affinity of antibodies, and association with serum lipoproteins have made detection of LPS and similar lipoglycans difficult targets for antigen specific assays [19,28,30,31]. Detection of the O-ag with classical methods such as latex agglutination or immunomagnetic separation utilize cross reactive polyclonal antibodies, which can lead to misidentification of the serogroup [32–36]. Enzyme-linked immunosorbent assays for detection of both LPS and O-ag serogroup identification have also been developed, but require extensive sample preparation, multiple antibodies, and yet suffer from non-specific interactions of the antibodies [30,37–41]. Polymerase chain reaction is also a method for detecting the specific LPS transport and polysaccharide biosynthesis genes. However, cross reactivity between specific genes of particular serotypes has been noted [42–44], leading to misidentification of those serotypes. Additionally, residual nucleic acids can indicate false positive results due to the presence of non-viable bacteria in samples [45].

Thus there is a need to improve current detection methods for identification of LPS O-ag. It has been well documented that amphiphiles, like LPS, interact both with lipid components of artificial membranes, as well as host serum-binding proteins [13,19,46–50]. Our team has previously explored the amphiphilic biochemistry of biomarkers such as phenolic glycolipid and lipoarabinomannan, and developed a tailored method, membrane insertion, for their detection [47,48,51]. Previously, we have reported on the detection of lipoarabinomannan using membrane insertion and sandwich immunoassays, and characterized the interaction of the amphiphile with lipid bilayers by atomic force microscopy (AFM) [47,48,51–53]. Our approach utilized a waveguide-based optical biosensor platform that was developed specifically for the ultra-sensitive detection of biomarkers [54,55]. This platform uses single mode planar optical waveguides functionalized with a lipid bilayer inside a flow cell to facilitate detection through the use of evanescent sensing and a fluorescently conjugated antibody [54–58]. This technique is based on the principle of exponential decay of the evanescent wave away from the surface of the waveguide material, which results in an excitation field that extends only 200 nm from the surface of the waveguide. Therefore, only samples and fluorophores within the evanescent field are illuminated by incident light. This minimizes background signal, thereby increasing the signal-to-noise (s:n) ratio of excited antibody-fluorophore conjugates bound to antigen at or near the surface of the waveguide. Waveguides are functionalized with supported lipid bilayer assemblies. Upon exposure to the amphipathic biomarker, the hydrocarbon tails passively diffuse through the aqueous matrix, and associate with the lipid bilayer, eliminating the need for capture antibodies [48,51]. In this manuscript, we show waveguide-based membrane insertion assays for detection of LPS O157 in ground beef lysate. Also presented are membrane insertion assays for detection of LPS from other serogroups, demonstrating broad applicability of this platform. For detection of LPS, this method helps to minimize exposure of conserved lipid A epitopes to cross reactive antibodies, while maximizing exposure of the highly specific O-ag to detection antibodies. Due to the heterogeneous nature of LPS, the inability to determine an accurate molecular weight or conformation of the antigens restricts the quantitative capability of assays for whole LPS. This is not a limitation of the assay or the platform, rather a critical issue with the detection of entire amphiphilic moieties by any methodology. Membrane insertion offers a reliable and direct strategy for the detection of amphiphilic targets in complex backgrounds with minimal sample preparation at high s:n levels and low (μg/mL) limits of detection. Detection of amphiphilic biomarkers is important for many pathophysiological measurements and in the study of host-pathogen biology, in addition to food safety.

To identify, describe, and delineate assay parameters, we have used biophysical methods to characterize the interaction of LPS with lipid bilayers. Lipid bilayers have been previously used to study the interactions of LPS in simple biomimetic systems [46,59]. Recent work from our team demonstrated LPS-induced deformations in 1,2-dioleoyl-sn-glycero-3-phosphocholine (DOPC) lipid bilayers based on ionic conditions [10]. These findings raised questions on the dynamics of the interaction of amphiphilic LPS with bilayers in membrane insertion assays. Since the detection antibodies would bind to the open glass substrate caused by hole formation, a high signal would result, which in the given scenario could be an effect of hole formation. In this manuscript, we address that question by devising a flow cell mimetic chamber to explore the interactions of LPS with lipid bilayers at conditions synonymous with our detection assays. Finally, we examine LPS-lipid bilayer dynamics using multiple serogroups of LPS to determine if the variable O-ag structure of the molecule affects the interactions with lipid bilayers, and explore the relevance to detection assays and the study of host-pathogen biology. Thus, we report membrane insertion as a reliable method for detection of entire LPS. The biochemistry of the target should be considered in all scenarios of detection as many factors can influence LPS micelle conformations and antigen presentation.

## Results and Discussion

### Detection of LPS with Membrane Insertion

**Fig 2:**
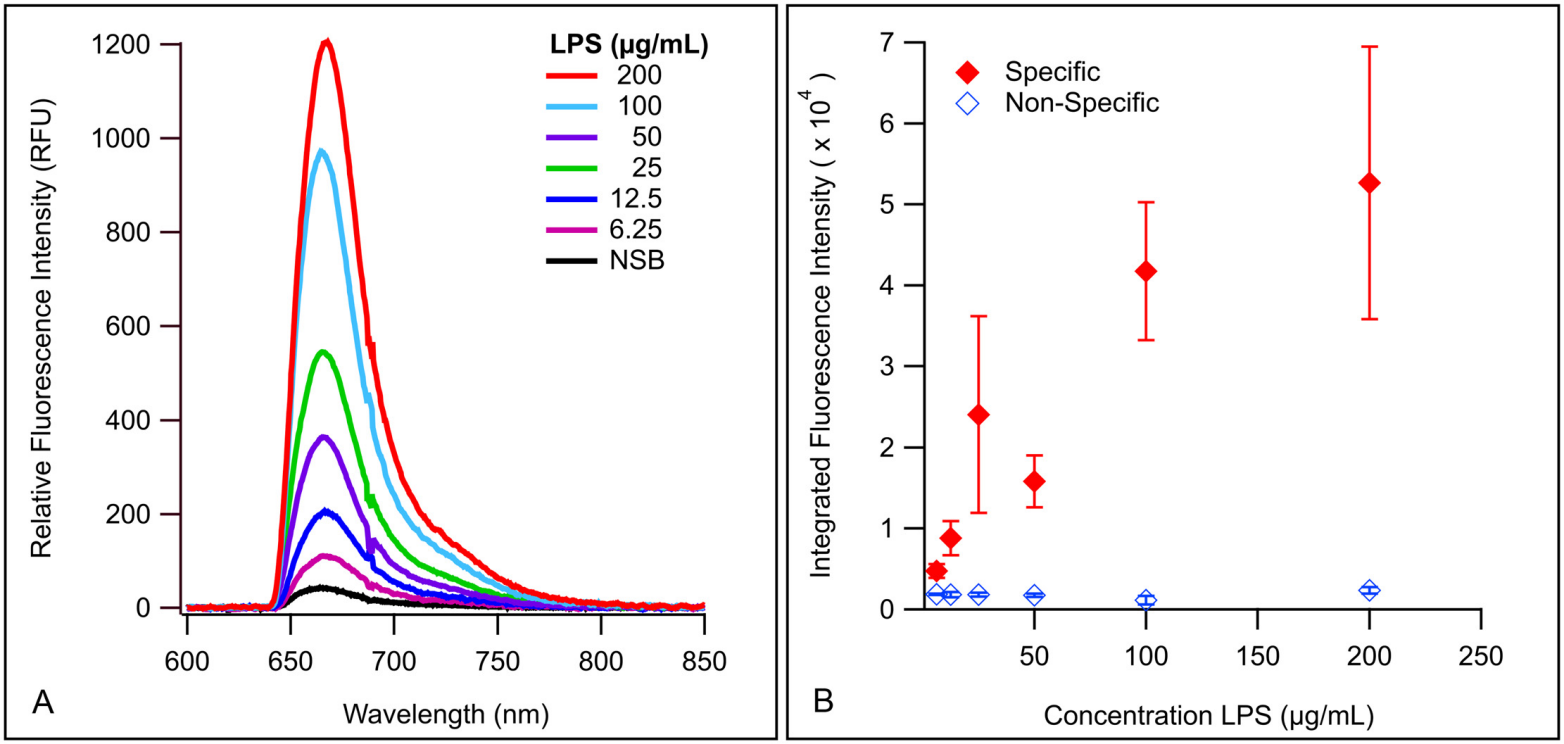
Membrane insertion for detection of LPS O157. (A) Spectral curves demonstrating detection of various concentrations of LPS O157. (B) Integrated values of spectral curves plotted as single points with standard error of the mean. Closed diamonds indicate averaged integrated signal intensity, and open diamonds are integrated NSB.

To determine the concentration range over which LPS can be reliably detected by membrane insertion, assays of LPS O157 were performed a minimum of three times over a concentration range of 6.25–200 μg/mL LPS (Fig 2A), using polyclonal antibody (pAb) anti-*E*. *coli* O157 (O157) labeled with Alexa Fluor^®^ 647 (af647) as the detection antibody (pAb O157-af647). The limits of detection (LoD) for LPS O157 were calculated to be 4.80 μg/mL using Eq (1) with the specific signal intensity values from the lowest concentration. The results indicated that membrane insertion consistently detects a broad concentration range of LPS with low non-specific binding (NSB) of the reporter antibody. However, the detection trend is non-linear (Fig 2B) with larger variability at higher concentrations. This lack of linearity is expected, and can be explained by the biochemical properties of amphiphilic LPS which significantly affect the size and conformational presentation of the molecule. For one, LPS will present in a micellar conformation in aqueous solutions [17,60–63]. Beyond the critical micelle concentration (CMC) of LPS O157, the amphiphile would exist both as monomers and aggregates [64,65], making repeatable quantitation challenging [64]. LPS micelles can further vary based on the size of the O-ag chains, which can be full-length, truncated, or absent entirely, depending on bacterial strain and growth phase [17,60,66]. Furthermore, LPS can also present in different shapes of micelles, such as lamellar, cubic, and hexagonal inverted structures [67–70], which are dependent on antigen structure, pH, ion concentration, solution composition, and temperature [10,17,62,71]. All of the above factors contribute to the size or shape of the micelles, and influence the binding availability of epitopes for detection, which in turn affects the inter-assay variability (Fig 2B). While reasonable efforts to control for the size of micelles in the preparations was taken (e.g. extended bath sonication [72] during testing of serogroup and beef lysate assays), we cannot be certain that LPS micelles in our assay systems are homogenous. This biochemical variability has limited the quantitative measurement of amphiphilic biomarkers in general [51,73]. Lastly, due to the stability of endotoxin [68,74] we cannot entirely discount the potential of endogenous endotoxin that may have been present on glassware, either from previous assays or other environmental bacteria, even though rigorous cleaning procedures were employed. This is also a relevant concern in beef lysates. We therefore only demonstrate the concentration range over which LPS can be reliably and repeatedly detected using membrane insertion. Membrane insertion is not intended to provide a quantitative measurement of concentration, but to accurately detect LPS with minimal sample processing in complex samples such as beef lysates. To determine that the variability between assays was caused by the variable nature of LPS, we employed rigorous statistical analysis of the data. Statistical regression analysis of the uncorrected data sets and the residuals (Tables A-C in S1 File) from multiple experiments demonstrate that factors such as antigen (LPS) concentration, choice of waveguide, power coupled into the waveguide, non-specific interaction of the antibodies with the lipid bilayer, and other systematic parameters do not account for the large deviations seen in detecting specific concentrations of LPS. The only significant factor resulting from the analysis is the antigen itself, though we also saw some significance associated with a single waveguide (Table C in S1 File). This suggests that variations in the CMC of the amphiphile, due to the heterogeneous nature and other biophysical properties, affect the interaction of LPS with the lipid bilayer and the detection antibody. This is further substantiated by measurements of protein binding on the same instrumentation in this and previous studies that do not present with such variability. Therefore, we conclude that the variability in signal at specific concentrations is primarily dependent on the conformation of the LPS antigens, and not variability associated with the detection platform, methods, or other reagents.

**Fig 3:**
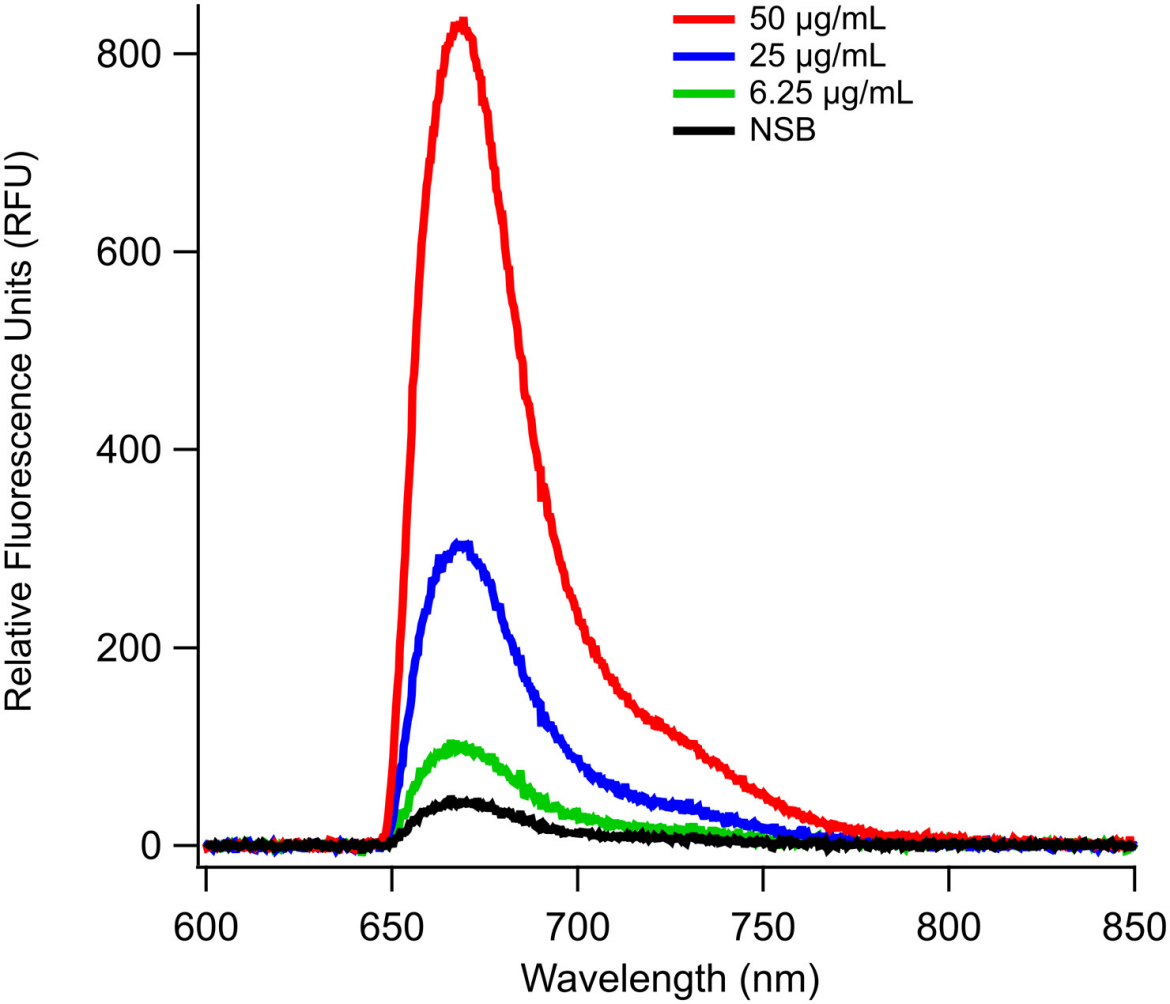
Concentration dependent detection of LPS O157 in 1 mg/mL beef lysates. Detection of LPS in beef lysates shows an increase in signal to noise ratios as compared to those seen in the benchmark assay.

To assess application for detecting contamination in food products, assays were performed in a complex sample matrix (e.g. beef lysate). The ability to detect LPS in beef products has historically required extensive processing and dilution of samples, and has only yielded information about endotoxin contamination with no clues as to pathogen virulence [75,76]. However, membrane insertion facilitates detection of whole, intact LPS, and discrimination of the O-ag present within the samples, which facilitates bacterial serotyping. Membrane insertion assays were performed in 1 mg/mL ground beef lysate at three concentrations over the range of 6.25–50 μg/mL LPS O157 (Fig 3). LoD for this assay was calculated to be 4.2 μg/mL LPS O157. The ratios between specific signal and NSB (s:n) at 6.25 μg/mL (~4), and at 50 μg/mL (~27) are comparable, albeit slightly higher, to the those seen in the benchmark assay (Table 1). However, the ratio at 25 μg/mL (s:n~10) was lower than that observed in the benchmark assay (Table 1). Despite this, the LoD for both assays are comparable (4.8 μg/mL *vs*. 4.2 μg/mL, benchmark and beef lysate respectively). The changes in the presentation and micelle properties of the antigen in complex physiological backgrounds can account for these observed differences in s:n ratios. We attribute the increased signal at 6.25 and 50 μg/mL to the possibility that LPS is known to associate with lipoproteins [13,19,47,48,53], such as low-density and high-density lipoproteins (LDL and HDL respectively), in serum and muscle tissue [77]. Since these lipoproteins carry amphiphiles and can insert them into membranes [50,78], it is possible that HDL and LDL are serving to insert monomeric LPS or LPS-lipoprotein complexes into the DOPC lipid bilayers which could serve to increase detection of O-ag. HDL is a critical factor for both treatment and prognosis of septic patients [79] because of its ability to shuttle amphiphilic LPS in hosts. No data is readily available on the CMC of LPS O157, however, it is reasonable to assume it to be somewhat similar to the CMC of LPS O111:B4 (22 μg/mL) [17]. This means that at 6.25 μg/mL, LPS would be present mostly as a monomer, and above 25 μg/mL, aggregates would be the primary conformation. At 25 μg/mL mL the change in the s:n ratio between the two assays could also be caused by the difference in solution composition between the beef lysate and benchmark (PBS) assays, which could affect micelle conformation. Additionally, the protein matrix of the beef lysate could be providing an additional blocking effect, which could increase the s:n ratio. It is tempting to speculate about the conformation of LPS at this specific concentration. This is especially important to consider when detecting multiple subtypes of LPS in complex matrices. Since conformation will vary slightly between different LPS antigens, the enhanced s:n ratios we see in the beef lysate will aid in the detection of multiple serogroups of LPS associated with STEC. Finally, the epitopes recognized by the detection antibodies, and their presentation can change significantly depending on the micelle conformation of LPS, which may contribute to the variability. In all instances, (e.g. benchmark, serogroup, or beef lysates) triplicate repeats of LPS membrane insertion assays demonstrated reliable results with μg/mL sensitivity within two hours. Thus, several factors can affect variation in measured detection signals of intact amphiphilic biomarkers such as LPS, and should be taken into account for the design and evaluation of diagnostic assays as well as the understanding of host-pathogen biology.

**Table 1:** Signal to Noise Ratios of LPS Membrane Insertion Assays.

	Signal:Noise Ratios	
LPS μg/mL	benchmark	beef lysate*
6.25	2.6	4.1
12.5	5.2	--------
25	13.4	9.8
50	8.8	26.9
100	23.3	--------
200	29.5	--------

*LPS O157 was tested at 3 concentrations in beef lysates

**Fig 4:**
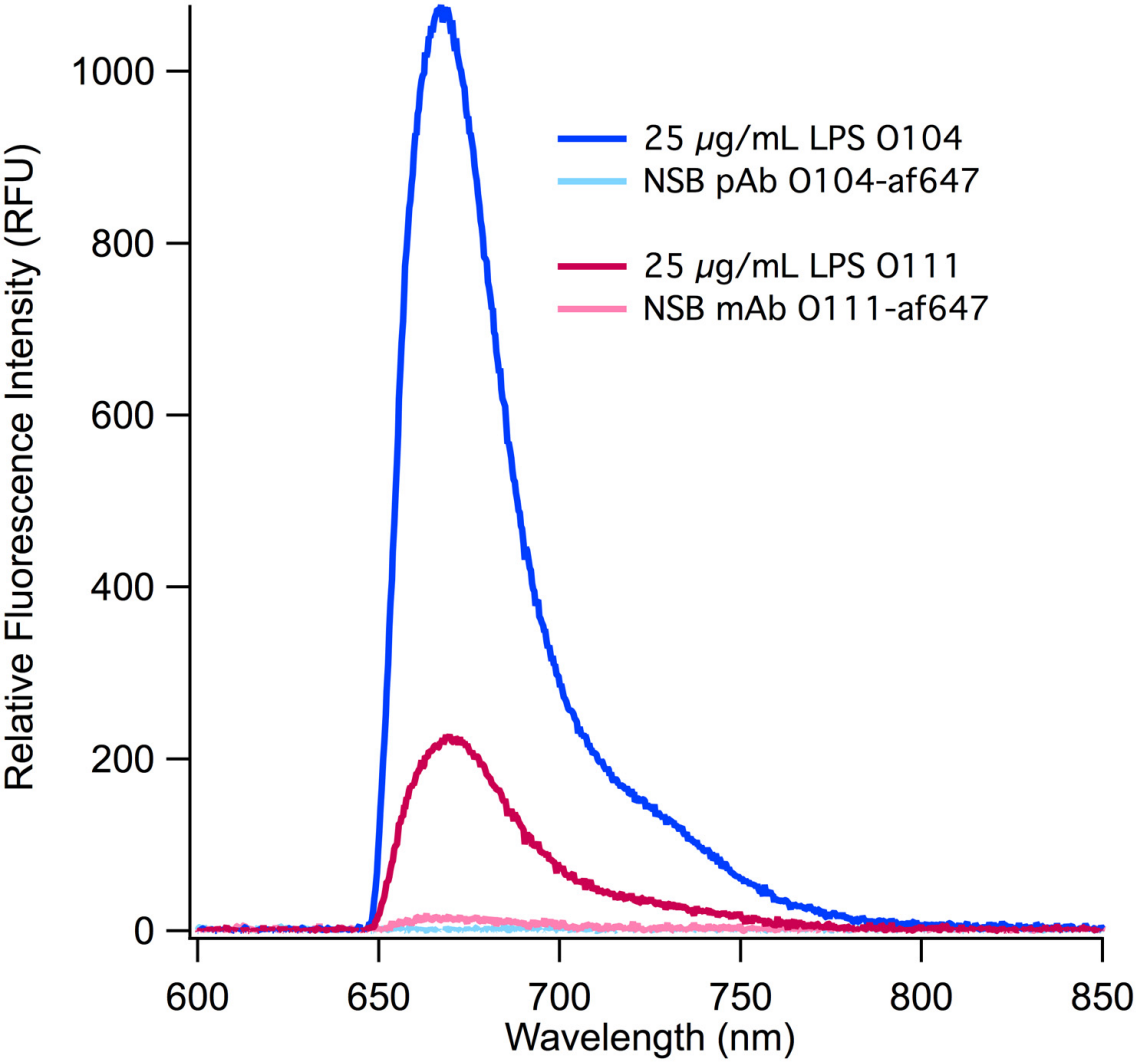
O-ag targeted detection of LPS. Using membrane insertion, two types of LPS were detected using their complement antibodies, polyclonal anti-*E*. *coli* O104 (pAb O104) and monoclonal anti-*E*.*coli* O111 (mAb O111) labeled with af647.

To demonstrate the broad applicability of membrane insertion assays, we tested LPS from other pathogenic *E*. *coli* (LPS O104:H4 and LPS O111:H11) using af647 labeled detection antibodies targeted against the specific O-ag (Fig 4), Sensitive detection is demonstrated in both cases with LPS O104 demonstrating a significantly higher (s:n~39) response than LPS O111 (s:n~6). This difference can largely be attributed to the sensitivity of the respective antibodies [36]. Due to the large difference in s:n ratios in these assays, the limits of detection also demonstrate the same pattern (0.77 and 7.36 μg/mL respectively). This is due in part to the low NSB of both antibodies, but also the specificity of the antibodies for their specific epitopes. Both LoDs fall within the reported range for physiologically relevant concentrations of LPS [78]. The LoDs we report are also an order of magnitude lower than those demonstrated by Rangin *et al*. [80,81] (2.2 mg/mL) when they reported specific detection of LPS in their benchmark assays using polydiacetylene liposome sensors. Our benchmark detection limit is also lower than that reported by Nieradka *et al*. [82], (50 μg/mL), who used self assembled monolayers to discriminate between LPS from different strains of *Hafnia alvei*. We observed a much lower variability between the assay replicates (S1 Fig) as compared to the benchmark assay at 25 μg/mL LPS. We attribute this primarily to the increased sonication time during antigen preparation that was implemented here, but also acknowledge that the O-ag of these LPS subtypes are much different from O157 and therefore may be more homogenous at this concentration. We would like to iterate that membrane insertion is the first to detect the O-ag of intact amphiphilic LPS directly in beef homogenates, as there is, there are not ideal comparisons for assay sensitivity and performance. The advantage of this method is not simply the sensitivity, but the ability to measure the entire moiety, which has significant physiological relevance.

A key factor that affects performance of any antibody-based assay is the sensitivity and specificity of the antibody being used. In membrane insertion, the amphiphilic antigen is presented partitioned into a lipid bilayer, which mimics the physiological presentation of such antigens *in vivo*. The antibody targeting LPS O104 is a polyclonal, extracted from an animal immunized with whole bacteria, and likely is more suitable for recognizing LPS when presented in a lipid carrier interface. In contrast, the antibody against LPS O111:H11, is monoclonal (mAb), and was raised *in vitro*. Also, polyclonal antibodies have multiple paratopes that can bind several different epitopes on the antigen, as compared to mAbs that target a single epitope. The source animal for antibodies may also play a large role in antibody affinity and specificity, since it has been demonstrated that different animal types exhibit varied levels of sensitivity to LPS [11,83], which would affect antibody expression. The above factors in antibody specificity and sensitivity are not unique to the two that are discussed here or to the membrane insertion approach, but indeed should be considered in the development of all assays involving detection antibodies.

### Imaging LPS-Lipid Bilayer Interactions inside a Flow Cell

**Fig 5:**
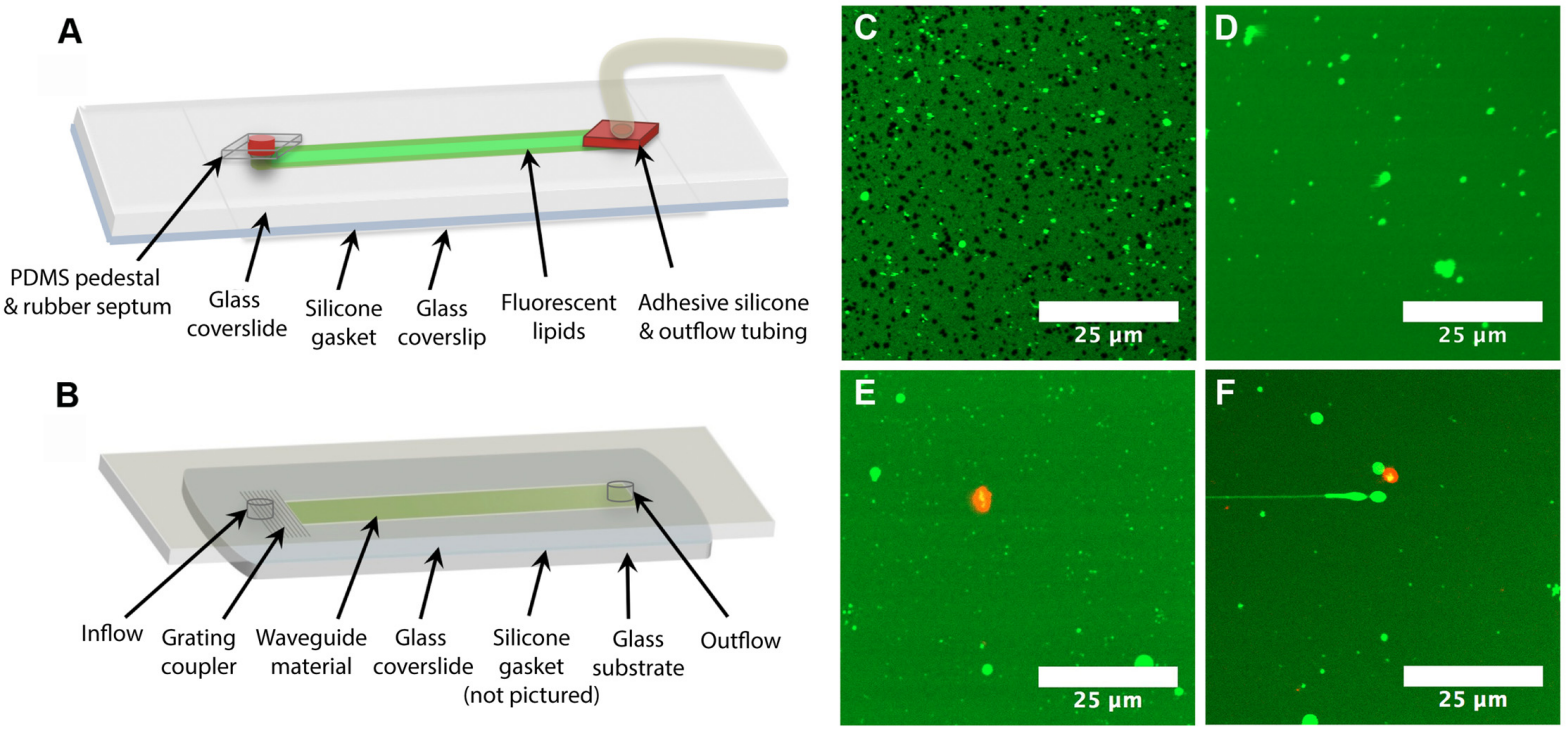
Assay performance inside flow cells. Schematics of (A) imaging flow cell and (B) waveguide flow cell. Major differences between these two flow cells include replacement of the waveguide glass substrate with a thin glass coverslip for imaging, and the addition of PDMS and silicone pedestals to create an airtight environment to preserve lipid integrity throughout an assay. (C) 100 μg/mL and (D) 50 μg/mL LPS O111:B4 incubated with BODIPY labeled DOPC lipids. Composite images of (E) 50 μg/mL and (F) 25 μg/mL LPS O157 bound by fluorescently labeled pAb O157-af647.

We have used fluorescence microscopy [10] as a tool to characterize amphiphile-lipid interactions, thereby building more robust membrane insertion assays for these difficult antigens. Previously, we have shown that LPS O111:B4 can form holes in supported lipid bilayers [10,84] using fluorescence microscopy. It therefore became imperative to determine whether hole formation was a limitation of LPS membrane insertion assays. To investigate this, we developed an imaging compatible flow cell model (Fig 5A) that replicated the internal dimensions and functional surfaces of the flow cell used in our waveguide-based assays (Fig 5B). This model enabled direct imaging of lipid bilayers, as well as the specific binding of the fluorescent antibodies to LPS (Fig 5C–5F). We investigated the effects of LPS O111:B4 (Fig 5C and 5D) and LPS O157 at 100 μg/mL, 50 μg/mL, and 25 μg/mL (Fig 5E and 5F), under the same conditions as the waveguide assays. We found that with LPS O157, the lipids maintained excellent lateral fluidity (S3 Fig) and there was no hole formation in the bilayers at any of the tested concentrations, thereby eliminating our concerns. LPS O111:B4, on the other hand, formed holes in lipid bilayers (Fig 5C) within the flow cell, but only at higher concentrations of antigen (>50 μg/mL) (Fig 5D). No hole formation was observed at lower, more physiologically relevant concentrations of LPS (Fig 5D). We were also able to generate composite images of the fluorescent lipids and the specific binding of pAb O157-af647 (Fig 5E and 5F) at localized spots within the flow cell for LPS O157. As demonstrated by the lack of overall red fluorescence (S4 Fig) in the images, the NSB of the antibody is quite low, while the specific binding intensity is saturated at localized positions. This data supports the low NSB signals seen in the membrane insertion assays, and serves as visual confirmation of antigen behavior.

### Imaging LPS Subtypes on Glass Slides

**Fig 6:**
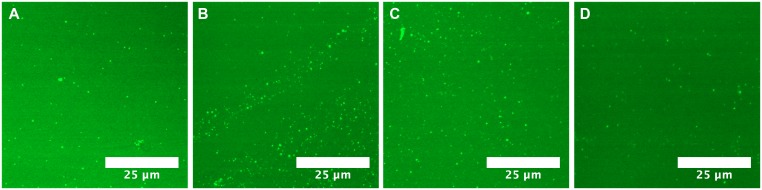
Imaging LPS O157 with lipid bilayers. (A) Bilayer prior to incubating with LPS O157. 50 μg/mL LPS (B) O157, (C) O104, and (D) O111:H11.

Due to the differential effects we saw between LPS O111:B4 and LPS O157, we evaluated the effect of 50 μg/mL LPS from various serogroups (O26, O45, O103, O104, O111, O113, O121, and O145) on open cover slides to observe membrane dynamics. LPS is an indicator of bacterial virulence, which in turn varies significantly between serotypes. These experiments were critical to determine whether other subtypes of LPS interacted differently with lipid bilayers and would therefore limit the capability of membrane insertion assays. Surprisingly, no membrane deformation was observed in any of the LPS serogroups (Fig 6 and S5 Fig), except the positive control, LPS O111:B4 (S5 Fig) [10]. The variability between these sub-types of LPS, and the difference in interactions with a simple lipid bilayer, are intriguing. Since the structure of the O-ag chain affects the CMC of LPS [17], the size and shape of the micelle produced in an aqueous medium can be different between LPS subtypes. Additionally, differences in O-ag structure combined with possible chemical signature differences in the core polysaccharide of LPS [68] between strains could contribute to a variable charge distribution in the LPS [69,70]. This, in turn, could affect the delamination of the lipid bilayer by LPS micelles. Lastly, there is the potential for capsular K polysaccharide antigens to be co-expressed in these different preparations of LPS [85]. The differential effect seen when using different types of LPS is a key indicator that small changes in biochemistry and structure can have a large impact on the interface between LPS (and other amphiphiles) and lipid bilayers. In other independent research we are currently exploring the effect of other environmental factors such as complex lipids, temperature, and pH on hole formation with different serogroups of LPS, to be reported in future studies.

Here we have shown that LPS behaves in dramatically different ways under different conditions. We demonstrated that it can be sensitively detected in complex beef lysate samples using membrane insertion with higher s:n ratios than those seen in the benchmark assays, which highlighted the important roles of LPS binding proteins (from the lysate) and solution composition, in the assay behavior. We also noted that the concentration dependent micelle properties of LPS in aqueous media, affects the interaction with bilayers at specific concentrations, most notably around a published CMC value for LPS [17], and at higher concentrations where LPS is known to form supramolecular aggregates [69], therefore causing the assay to be non-linear. We also saw a concentration dependent effect in LPS-induced hole formation when we incubated LPS O111:B4 in the flow cell mimetic where we saw no deformities of the membrane at lower concentrations. Additionally, the absence of hole formation when using different types of LPS is also a key indicator that the behavior of very similar amphiphiles can be dramatically different. This data complements our previous results which demonstrate that simply changing from a monovalent to a divalent cation in the buffer solution can also effect amphiphile behavior when incubating with lipid bilayers [10]. Together, the results listed above indicate the critical importance of controlling conditions to manage amphiphile behavior, especially when interfacing LPS with bilayers. The implications of these conclusions do not escape us, as many studies that use LPS do not take necessary steps to control amphiphile behavior, and may therefore achieve both unexpected and difficult to repeat results. These results may also help explain the difference in biological activities of different serogroups of LPS, which may not be solely attributable to the structure of lipid A [16]. Since LPS is globally used as an immune stimulant and a key indicator of bacterial infection, the continued study of this molecule is critical for understanding host-pathogen interactions and developing better amphiphilic detection platforms.

These studies demonstrate the challenges associated with the measurement of amphiphilic biomarkers such as LPS. The biochemistry of LPS causes altered behavior of the molecule when small differences are made to its environmental system. This is an especially important consideration to take into account not just for LPS, but for all amphiphiles which may be indicators of infection or disease. Previous studies reporting the poor sensitivity of assays for the direct detection of LPS and other biomarkers in aqueous mileau, such as blood, have ignored their amphipathic biochemistry. With this manuscript and others, we hope to unravel the challenges associated with the detection of such biomarkers in clinically relevant samples, and develop strategies to overcome them effectively in the future.

## Materials and Methods

### Materials

Lipopolysaccharides from six strains of non-O157 STEC (DEC10B [O26:H11], B8227-C8 [O45:H2], MT#80 [O103:H2], 0201 9611 [O111:H11], MDCH-4 [O113:H21], DA-37 [O121:H21], GS G5578620 [O145:NM], and TY-2482 [O104:H4]) were selected and prepared by hot phenol extraction and tested for antigen activity as we have previously described [36]. LPS O157:H7 was purchased from List Biological Labs (Campbell, CA), and LPS O111:B4, bovine serum albumin (BSA), Dulbecco’s phosphate buffered saline (PBS), Ethylenediaminetetraacetic acid (EDTA), and potassium chloride were from Sigma Aldrich (St. Louis, MO). Polyclonal antibody anti-*E*. *coli* O157 was from LifeSpan Biosciences (Seattle, WA). pAb *E*. *coli* O104, as well as monoclonal antibody for *E*. *coli* O111 were from Abraxis Inc. (Warminster, PA). 1,2-Dioleoyl-sn-glycero-3-phosphocholine (DOPC) and 1,2-dioleoyl-*sn*-glycero-3-phosphoethanolamine-N-(cap biotinyl) (sodium salt) (cap-Biotin) were obtained from Avanti^®^ Polar Lipids (Alabaster, AL). C5-BODIPY^®^ FL HPC (2-(4,4-difluoro-5,7-dimethyl-4-bora-3a,4a-diaza-s-indacene-3-pentanoyl)-1-hexadecanoyl-*sn*-glycero-3-phosphocholine) was purchased from Molecular Probes^®^ (Eugene, OR). Sylgard^®^ silicone elastomer kit (Dow Corning, Midland, MI) was used to pour a 90/10 mix of polydimethylsiloxane (PDMS). Alexa Fluor^®^ 647 labeling kits, UltraPure™ Glycerol, and HEPES were all from Life Technologies (Thermo Fisher Scientific, Grand Island, NY). Silicon oxynitrite waveguides were purchased from nGimat (Norcross, GA) and the functional surface of silicon dioxide was maintained by Spectrum Thin Films (Hauppauge, NY). Silicone gaskets for waveguide assembly were from Grace Bio-Labs (Bend, OR) and Secure seal spacers (9 mm diameter x 0.12 mm deep) were from Electron Microscopy Sciences (Hatfield, PA). Glass microscope slides, Gold Seal™ cover glass, and sucrose were purchased from Thermo Fisher Scientific (Rockford, IL). Epoxy was from Gorilla Glue, Inc., (Cincinnati, OH), and Simple Truth^®^ organic ground beef was purchased from the local Kroger Stores (Los Alamos, NM). All reagents were of the highest quality for their intended purpose.

### Waveguide Preparation

Single mode planar optical waveguides were cleaned and prepared as previously described [33,54,55,57,86,87]. In brief, the waveguides and coverslides were cleaned by bath sonication for 5 min each in chloroform, ethanol, then water. Waveguides and coverslides were dried under an argon stream and exposed to UV-ozone (UVOCS Inc., Montgomeryville, PA) for 40 min. Flow cells for immunoassays were immediately assembled using cleaned waveguides and coverslips which were bonded together by a silicone gasket with a laser cut channel in the center. Following assembly, the flow cells were injected with a preparation of lipid micelles, then incubated overnight at room temperature (RT), to facilitate vesicle fusion [57].

### Micelle Preparation

Micelles for waveguide membrane insertion experiments were prepared by probe sonication as previously described [33,54,55,86,87]. 2 mM DOPC and 1% (mol/mol) cap-Biotin were prepared by deposition of chloroform-dissolved lipids into glass tubes, and evaporation of solvent under an argon stream. Biotin incorporation allows for the evaluation of bilayer integrity at the conclusion of assays [51,57]. Lipids were rehydrated in phosphate buffered saline (PBS), stirred for 2 hours (hr) at RT, 120 revolutions per minute (rpm) on an orbital shaker, followed by 10 freeze-thaw cycles. Finally lipids were probe sonicated for 6 min (1.0 s pulse on/off, 15% amplitude) using a Branson ultrasonic generator.

Micelles for fluorescent imaging were prepared in a similar fashion as those for waveguide experiments with the addition of 0.5–1% (mol/mol) of C_5_-BODIPY FL HPC to serve as a fluorescent marker for imaging. Lipids in chloroform were vacuum dessicated overnight and subsequently prepared in PBS, followed by 6 freeze-thaw cycles and 10 minutes of continuous probe sonication (tip dia. = 3 cm, 12 watts) (Sonicator 3000, Misonix, Farmingdale, NY)

### Lipopolysaccharides, Beef Samples, and Antibodies

Except in the cases of concentration dependence assays, LPS stocks (5 mg/mL) were thawed and bath sonicated for 15 min, diluted to the working concentration in PBS and sonication was repeated prior to injection in the flow cell. For the benchmark assays on concentration dependence of LPS, the stocks were sonicated for 5 min, diluted to working concentration in PBS and resonicated for an additional 5 min prior to injection.

Ground beef was flash frozen in liquid nitrogen, and freeze-dried on a Schlenk line for 48 hr. Dried material was crushed using a mortar and pestle, then homogenized in lysis buffer (0.5 M sucrose, 10 mM HEPES, 25 mM KCl, 1 mM EDTA, 10% v/v glycerol, 5 mg/mL concentration) [33]. The suspension was alternately vortexed (30 sec) and bath sonicated (30 s) until large protein aggregates were eliminated. Samples were diluted to 1 mg/mL in PBS immediately before use. The beef homogenate was used as a negative control, in order to evaluate background fluorescence and assess antibody cross-reactivity with a crude matrix that simulates an actual test sample. Additionally we also spiked LPS directly into homogenates to determine detection capabilities in a beef sample [33].

Reporter antibodies for LPS were pAb anti-*E*. *coli* LPS O157 (pAb O157), pAb anti-*E*. *coli* LPS O104:H4 (pAb O104) and mAb anti-*E*.*coli* O111:H11 (mAb O111). All reporter antibodies were fluorescently labeled with Alexa Fluor^®^ 647 (af647) per kit instructions. Molar ratio of dye to protein was measured using a NanoDrop™ 1000 (Thermo Scientific) and calculated (3.68 for LPS concentration assays, and 7.37 for beef lysate assays) per Alexa Fluor^®^ kit instructions. Degree of labeling for pAb O104-af647 was 3.17 and that for mAb O111-af647 was 7. After labeling, antibodies were checked for activity using immunoblotting of 5 mg/mL LPS antigens onto nitrocellulose, and compared with immunoblotting results for antibodies prior to labeling.

### LPS Membrane Insertion Assays

In all cases, unless stated otherwise, membrane insertion assays were performed in triplicate (minimum number of repeats) using the same concentrations of antibody, method of LPS preparation, and incubation times. All volumes (sample, antibody, beef lysate) were 200 μL. Concentration dependent LPS insertion assays were using LPS O157 and 25 nM pAb O157-af647 as the reporter antibody. Flow cells were prepared as described and blocked for 1 hr with 2% (w/v) BSA, then rinsed with 0.5% BSA/PBS. Incident light from a 635 nm laser, (power 440–443 μW) was coupled into the waveguide using a diffraction grating. The response signal was adjusted for maximum peak intensity using a spectrometer (USB2000, Ocean Optics, Winter Park, FL) interfaced with the instrument and an optical power meter (Thor Labs, Newton, NJ) [33,54,55,58]. The background signal associated with the lipid bilayer and protein block was recorded after which the flow cell was incubated (90 min) with pAb O157-af647 to determine NSB between the antibody and the lipid bilayer. The flow cell was rinsed with 2 mL of wash buffer (0.5% BSA/PBS) after all incubations. LPS was incubated for 2 hr to allow maximal association with the supported lipid bilayer. Excess LPS micelles were removed by washing and the signal recorded. Subsequently, reporter antibody was incubated for 90 min and rinsed, and the specific signal associated with antibody bound to LPS captured on the bilayer was recorded.

Membrane insertion assays for serogroups of LPS were performed in triplicate at a concentration of 25 μg/mL, using pAb O157-af647 as the reporter antibody. This approach exploits the cross-reactivity of a polyclonal antibody to the conserved O-ag regions of different serogroups of LPS [36]. However, we raised the hypothesis that by use of antibodies specific for a particular LPS serogroup, we could potentially enhance the sensitivity and selectivity of detection by targeting the variable O-ag region. To evaluate this, LPS O104 was tested under identical conditions using 25 nM pAb O104-af647 as the reporter, and then compared to the signal using the non-specific pAb O157-af647. Additionally we also tested whether using mAb specific to the O-ag would increase the specific signal and tested LPS O111:H11 with its respective mAbs.

To determine NSB of the detection antibody with the beef lysate, a 1 mg/mL beef homogenate sample was prepared by diluting in PBS and incubating with the bilayer for 2 hr. NSB of the reporter antibody was assessed against the beef lysate after a 90 min incubation, and then LPS (6.25, 25, or 50 μg/mL) was spiked into beef lysate and incubated for 2 hr. Specific signal was recorded after 90 min incubation with the reporter antibody.

### Imaging Inside of a Flow Cell

Due to previous observations that LPS could induce hole formation in DOPC lipid bilayers, we investigated this mechanism as a possible limitation of membrane insertion assays. To accomplish this, we established a flow cell mimic to investigate the interactions of LPS with DOPC bilayers inside a flow cell of identical dimensions and functionalized surfaces as our waveguide biosensor (Fig 5A and 5B). For this, two holes were drilled into a glass slide and a 24x50 mm cover glass was used in place of the waveguide piece to allow imaging. Glass was cleaned in 30:10 sulfuric oxides for 40 min then rinsed repeatedly and bath sonicated 3 times (5 min/each) in deionized water. The flow cell model was constructed from the two glass pieces with the addition of an attached outflow tube and a rubber septum to allow buffer exchange. PDMS (90:10 elastomer:curing agent) was poured into plastic petri dishes to a final height of ~4 mm, allowed to cure, and then cut into a square (~1 cm x 1 cm). To create an injection port, a rubber septum was inserted into the PDMS when it was approximately halfway cured. For the fluid outflow port, a 2 mm hole was made in a 1 cm^2^ of self-adhesive silicone using a biopsy punch and tubing was inserted through the hole. PDMS and flow cell assembly was then exposed to UV-Ozone for 2 min after which PDMS/septum assembly and silicone were stuck to the glass slide and seams were sealed using epoxy. Epoxy was allowed to cure for 1 hr prior to deposition of 2 mM DOPC + 1% biotin + BODIPY^®^ labeled lipid micelles. Lipids were deposited into the flow cell, the outflow tube was clamped shut, and the apparatus was incubated O/N at 4°C in the dark. Flow cell was rinsed with 10 mL PBS and imaged on an Olympus IX-81 motorized inverted microscope with excitation provided by a 488 nm Argon ion laser and green filter set. Fluorescence recovery after photobleaching (FRAP) was used to confirm lateral fluidity of lipid bilayers. LPS membrane insertion assays were then performed in the same manner as the waveguide assays (duplicate repeats), with images recorded to determine hole formation (or lack thereof) under these conditions. In most cases, images were recorded at 1024 x 1024 pixels at a scan rate of 12.5 μs/pixel. FRAP was performed on 512 x 512 pixel frames, using 5x zoom, at a scan rate of 10 μs/pixel.

### Imaging LPS on Glass Slides

To determine differential interactions of various LPS serogroups on DOPC lipid bilayers, 9 mm secure seal spacers were adhered to clean glass cover slides and 2 mM DOPC + BODIPY^®^ micelles were deposited and incubated for 20 min as previously described [10]. Free lipid vesicles were rinsed away using 10 exchanges of PBS buffer (1 mL total volume) and then LPS was prepared and incubated with the bilayers for 20 min at RT, after which free LPS was rinsed away with 10 exchanges of buffer. A minimum of two replicates was obtained for each serogroup of LPS. Negative and positive controls (LPS O111:B4 and buffer, respectively) were run in parallel to each experiment. FRAP and fluorescence imaging was used to determine the effect of the LPS groups on the fluidity and conformation of the bilayers. Data was optimized for contrast and brightness using ImageJ 1.48.

### Data Processing

Resulting spectra from the waveguide biosensor was processed and graphed using Igor Pro 6.37. Due to NSB signals that were nearly equivalent to background values, the data for the membrane insertion assays of LPS O104:H4 and O111:H11 were not background corrected, and were integrated as raw spectral curves between 550 and 850 nm and then averaged. In all other cases, individual spectra replicates were integrated between the wavelengths of 550–850 nm, where the significant signal appears for detection with af647 and a long pass 647 nm filter, and then corrected for background noise levels. Integrated values were then averaged and used to calculate a s:n ratio. LoD were obtained by taking the average integrated NSB for all replicates in a set, determining the standard deviation (σ) of the replicates, adding 3σ, then multiplying by the sample concentration (μg/mL), and dividing by the integrated average specific signal for that concentration (see Eq 1).

$$LoD=\frac{\left(NSB+3\sigma )[Sample\right]}{Specific}$$ (1)

### Statistical Analysis

Linear regression was used to relate the logarithm of the raw integrated intensities according to LPS concentration (LPSc), waveguide ID (wg#), power coupled (power), and type of measurement (background (mBG), non-specific (mNSB), specific, and specific (mSP)). Analysis of variance (ANOVA) was then used to determine the significance of the variables at the 5% level, (Table A in S1 File). Subsequently, to explain the observed heteroscedasticity, we regressed the absolute value of the residuals from the previous regression analysis onto the same set of explanatory variables (Table B in S1 File).

Model selection was performed using Akaike information criterion to determine the significance of the variables. Absolute values of the residuals of the means for LPSc, wg#, and power were processed with regression analysis (Table C in S1 File) using the type of measurement as a covariate.

## Supporting Information

S1 Appendix Integration algorithm for spectral data processing. Short algorithm used in IgorPro to individually integrate the raw spectral waves from an Ocean Optics Spectrometer.
(PDF)

S1 Dataset Integrated spectral values and data processing method. Excel spreadsheet which contains all the integrated values of the spectral curves and how those values were processed to obtain limits of detection and signal to noise ratios. Data was integrated using IgorPro 7 and algorithm available in S1 Appendix.
(XLSX)

S2 Dataset Raw data of spectral curves. Excel spreadsheet that contains the spectra collected from a waveguide-based optical biosensor fitted with a USB 2000 Ocean Optics spectrometer. File contains 11 different tabs, and the replicates for each concentration or assay are contained within a single tab. Concentrations are clearly marked, and the waveguide number is written after each replicate number. E.g. N = 1/wg#.
(XLSX)

S1 Fig Integrated intensities of O-ag targeted detection of LPS. Spectra from Fig 4 were integrated and plotted to demonstrate the difference in values when using specific antibodies for detection. Error bars indicate standard error of the mean for the average of three replicates.
(TIF)

S2 Fig High concentration of LPS O157 in a flow cell. 100 μg/mL LPS O157 was incubated in the flow cell and rinsed. No hole formation was observed.
(TIF)

S3 Fig Lateral fluidity of bilayers after incubation with 100 μg/mL LPS O157 inside a flow cell. (A) Time lapse series of DOPC-BODIPY bilayers that were photobleached and showed lateral fluidity during recovery. (B) Intensity profile graph of the overall average intensity and the recovery of the photobleached region. Incubating with LPS O157 does not cause hole formation or effect fluidity of the bilayers.
(TIF)

S4 Fig Specific and non-specific binding of pAb O157-af647 inside a flow cell. (A) Composite 2 channel image of DOPC-BODIPY lipids and pAb O157-af647. White arrows indicate points of fluorescence intensity, and the white dotted line is the region of analysis graphed in D. Arrow 1 is a DOPC-BODIPY surface associated vesicle, and arrow 2 is specific binding of the reporter antibody. (B) Green channel of image A. (C) Red channel of image A. White arrows indicate points of non-specific binding. (D) Line intensity profile of dotted line in image A showing low non-specific binding and saturated intensity of the specific binding. Low NSB and high specific binding events allow for increased signal to noise ratios allowing sensitive detection of LPS membrane insertion.
(TIF)

S5 Fig Effects of multiple serogroups of LPS on lipid bilayers. (A-I) 50 μg/mL LPS O111:B4, O26, O45, O103, O104, O111, O113, O121, and O145 respectively.
(TIF)

S1 File Statistical results and tables. Presents a brief overview of the results obtained from each ANOVA and the regression analysis of residuals.
(PDF)
