# Supplementary material for: Membrane Insertion for the Detection of Lipopolysaccharides: Exploring the Dynamics of Amphiphile-in-Lipid Assays
Source: PLoS One. 2016 May 26;11(5):e0156295. doi: 10.1371/journal.pone.0156295 (PMC4881986; doi:10.1371/journal.pone.0156295)
Supplement: S1 Appendix — Short algorithm used in IgorPro to individually integrate the raw spectral waves from an Ocean Optics Spectrometer. (PDF) [file pone.0156295.s001.pdf]

Spectral Data Processing Algorithm

Igor Pro Procedure:
This simple procedure is designed to take columns of spectral data and take the area under the curve as a function of the change in wavelength. This takes advantage of a built-in Igor Pro 6.3 function called ‘*areaXY*’.

```
Function BIR5(W, B1, B2, B3, B4, B5, NSB, SP, index) //input function –
designed to intergrate input waves B1, B2, B3, B4, B5, NSB, SP where
ΔX is determined by the ΔW – usually about 0.3 nm
//The number of input background variables should be altered if using a data set
that has more or less responses recorded.

//Variables
Wave NSB, SP //non-specific binding and specific binding spectra
Wave B1, B2, B3, B4, B5 //background spectra
Wave W //wavelength range scanned
Variable index

Make /O/N = 100 NSB_int, SP_int, B1_int, B2_int, B3_int, B4_int, B5_int, ratio
//generates the empty waves to allocate the resulting values into
	//if processing larger data sets (greater than 100 values expected), the
size of these waves should be changed accordingly


NSB_int[index] = areaXY(W, NSB) //calculates the area under the curve of NSB
and using the wavelength as the limits of integration...trapezoidal method

//index is using an assigned value to direct results to a specific location in the
wave NSB_int – following commands are engineered in an identical fashion, thus
assigning values from one experiment to the same row in each wave

SP_int[index] = areaXY(W, SP) //calculates the area under the curve of SP and
using the wavelength as the limits of integration...trapezoidal method

B1_int[index] = areaXY (W,B1)
B2_int[index] = areaXY (W,B2)
B3_int[index] = areaXY (W,B3)
B4_int[index] = areaXY (W,B4)
B5_int[index] = areaXY (W,B5)


ratio[index] = SP_int[index]/NSB_int[index]

End
```
