# Supplementary figures and images for: Membrane Insertion for the Detection of Lipopolysaccharides: Exploring the Dynamics of Amphiphile-in-Lipid Assays
Source: PLoS One. 2016 May 26;11(5):e0156295. doi: 10.1371/journal.pone.0156295 (PMC4881986; doi:10.1371/journal.pone.0156295)

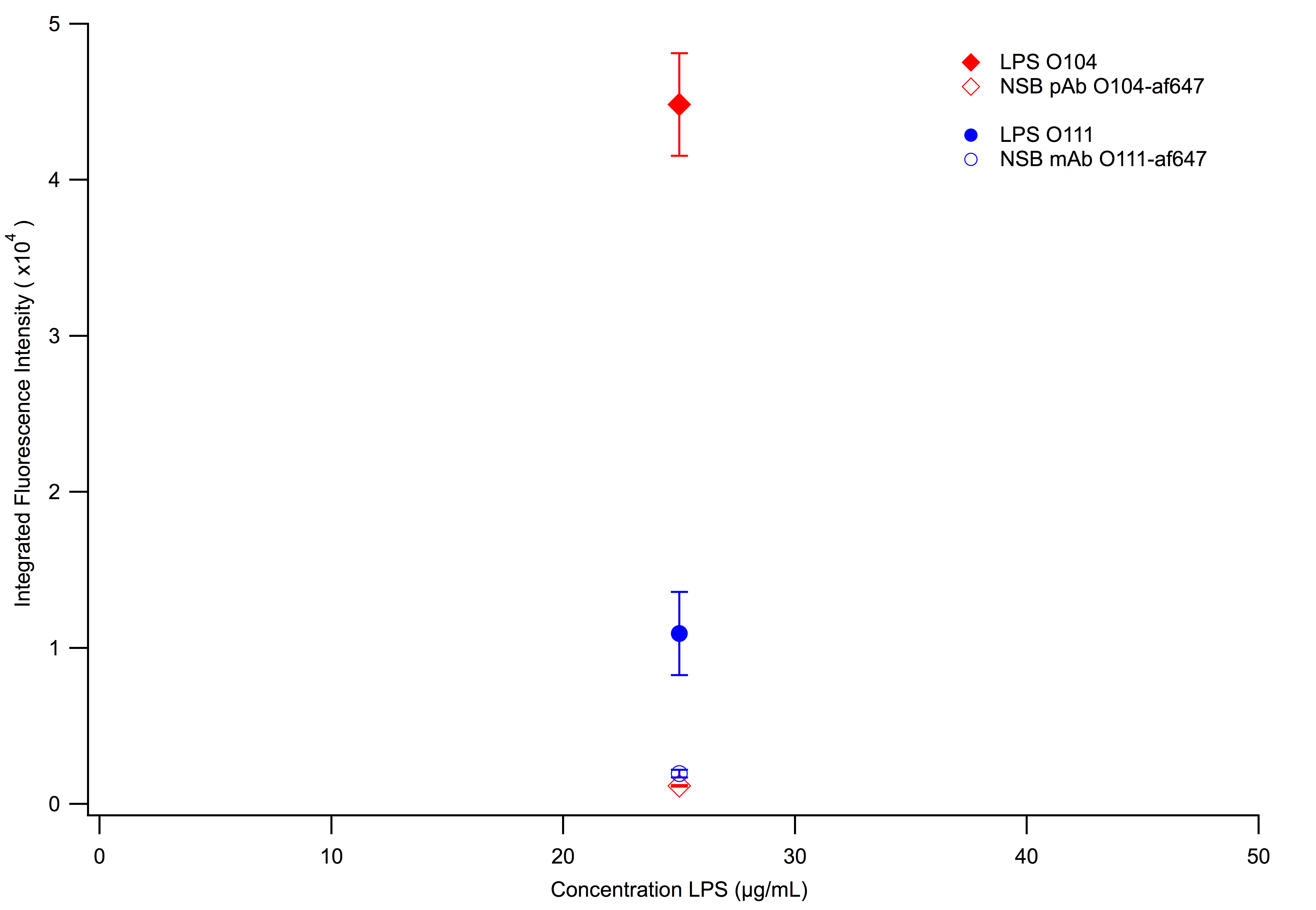

Supplement: S1 Fig — Spectra from Fig 4 were integrated and plotted to demonstrate the difference in values when using specific antibodies for detection. Error bars indicate standard error of the mean for the average of three replicates. (TIF) [file pone.0156295.s004.tif]

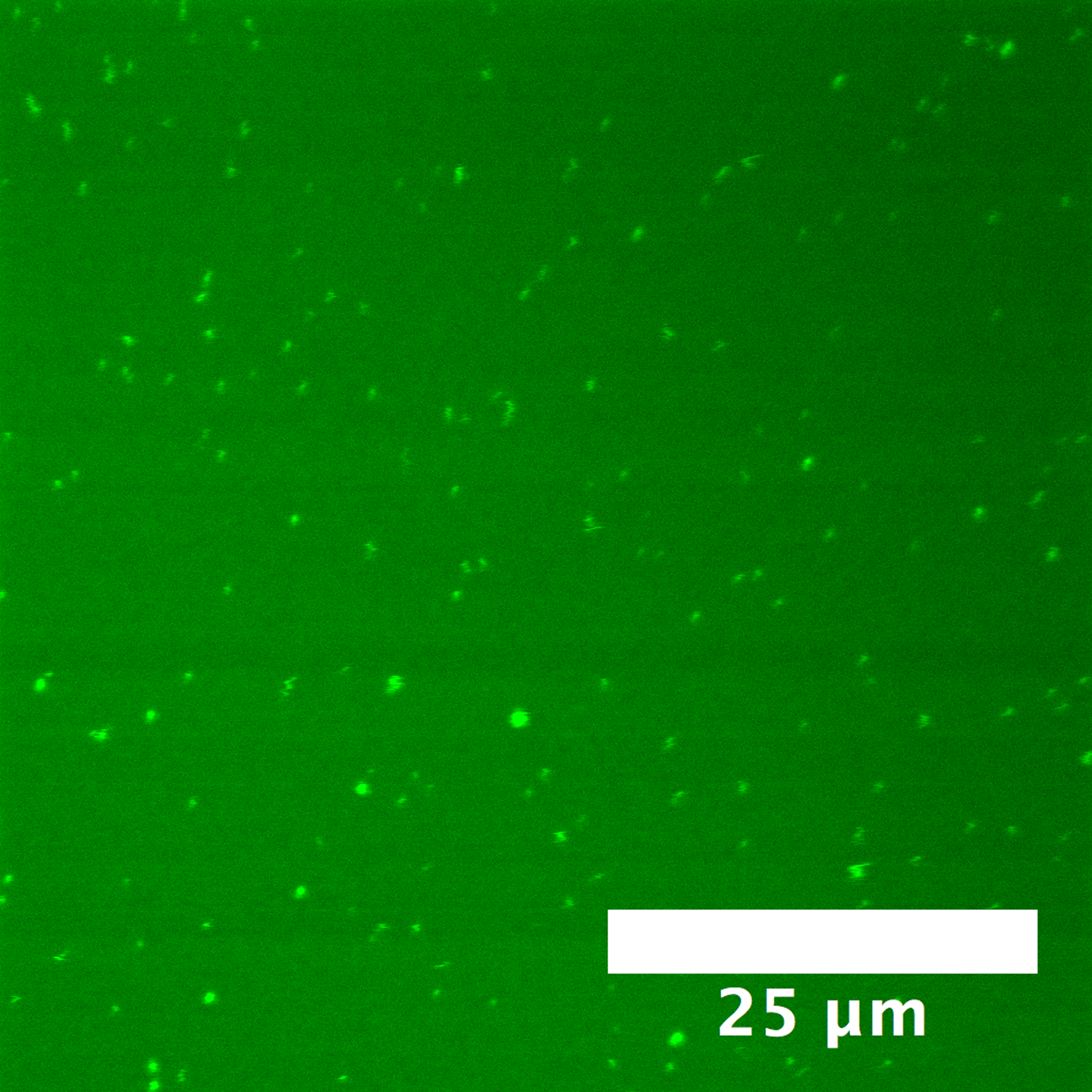

Supplement: S2 Fig — 100 μg/mL LPS O157 was incubated in the flow cell and rinsed. No hole formation was observed. (TIF) [file pone.0156295.s005.tif]

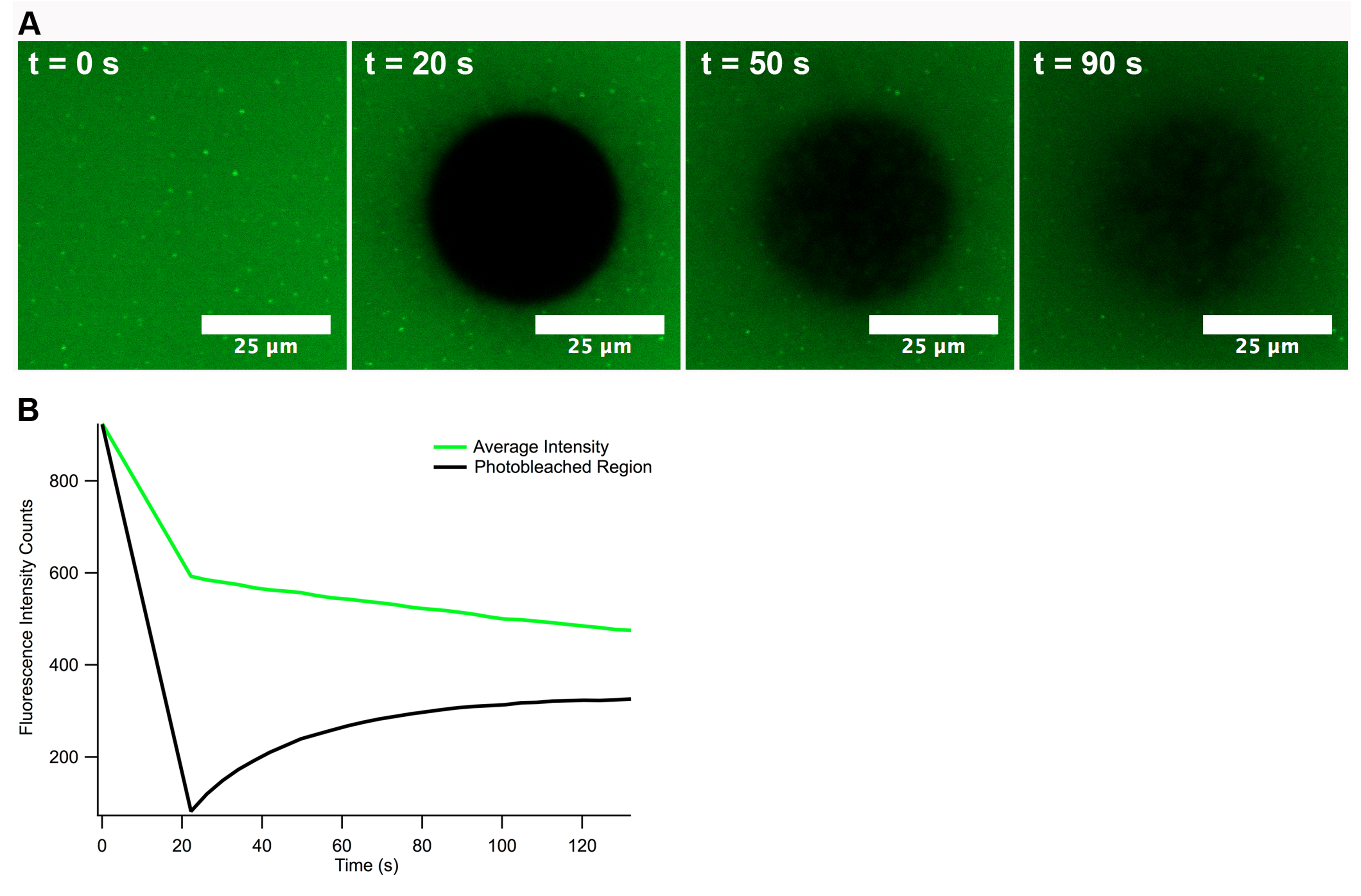

Supplement: S3 Fig — (A) Time lapse series of DOPC-BODIPY bilayers that were photobleached and showed lateral fluidity during recovery. (B) Intensity profile graph of the overall average intensity and the recovery of the photobleached region. Incubating with LPS O157 does not cause hole formation or effect fluidity of the bilayers. (TIF) [file pone.0156295.s006.tif]

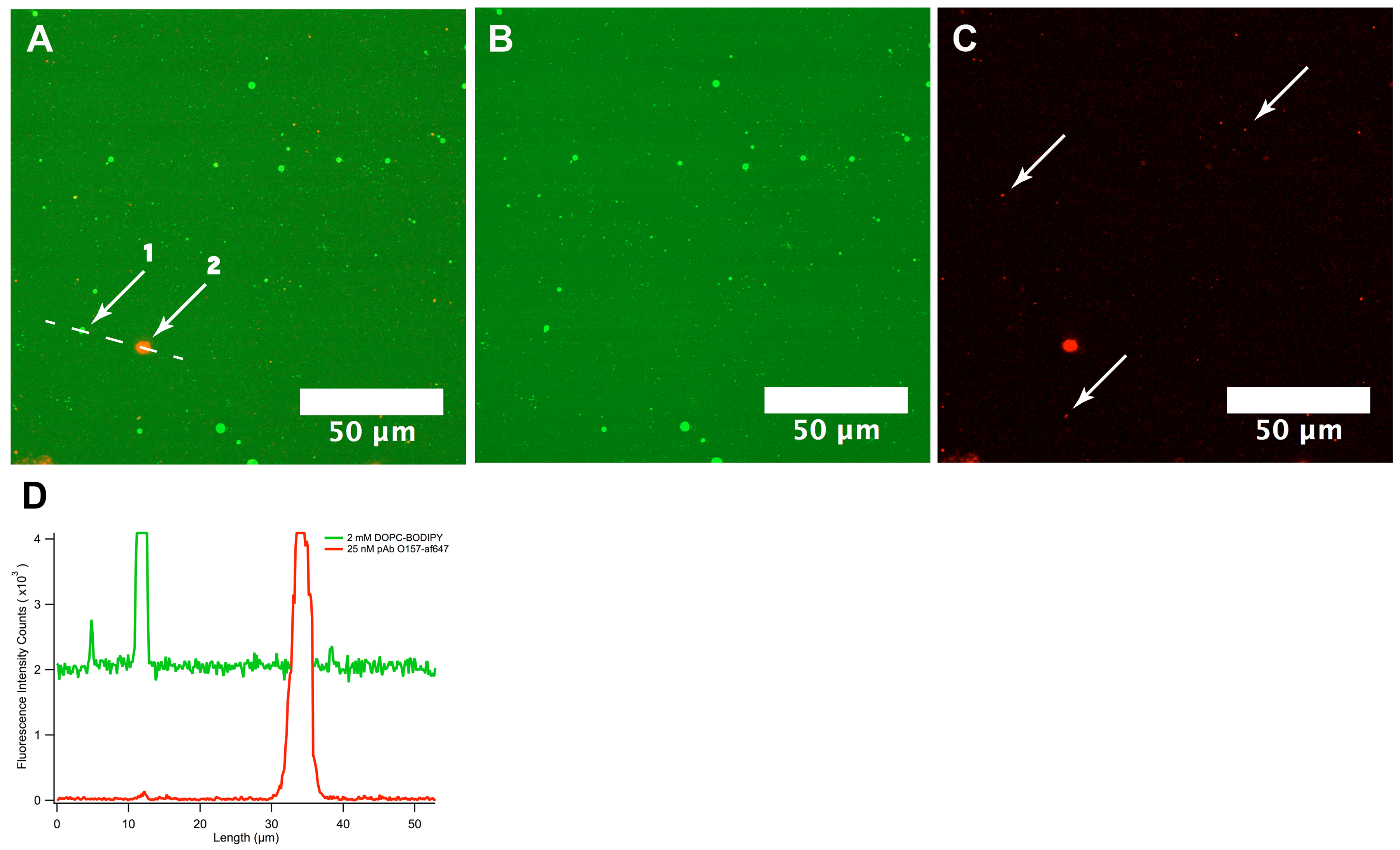

Supplement: S4 Fig — (A) Composite 2 channel image of DOPC-BODIPY lipids and pAb O157-af647. White arrows indicate points of fluorescence intensity, and the white dotted line is the region of analysis graphed in D. Arrow 1 is a DOPC-BODIPY surface associated vesicle, and arrow 2 is specific binding of the reporter antibody. (B) Green channel of image A. (C) Red channel of image A. White arrows indicate points of non-specific binding. (D) Line intensity profile of dotted line in image A showing low non-specific binding and saturated intensity of the specific binding. Low NSB and high specific binding events allow for increased signal to noise ratios allowing sensitive detection of LPS membrane insertion. (TIF) [file pone.0156295.s007.tif]

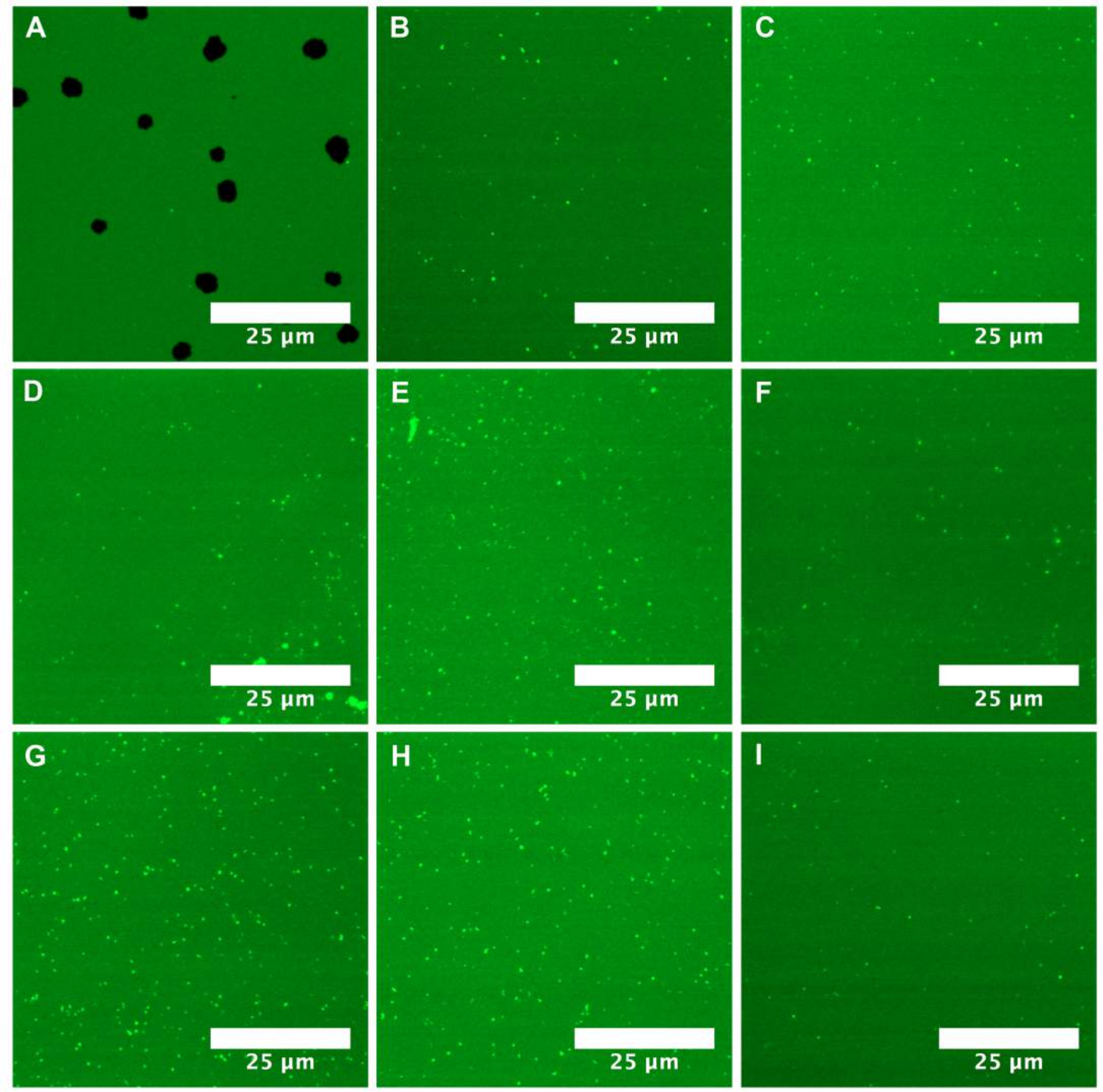

Supplement: S5 Fig — (A-I) 50 μg/mL LPS O111:B4, O26, O45, O103, O104, O111, O113, O121, and O145 respectively. (TIF) [file pone.0156295.s008.tif]
