# Supplementary material for: Membrane Insertion for the Detection of Lipopolysaccharides: Exploring the Dynamics of Amphiphile-in-Lipid Assays
Source: PLoS One. 2016 May 26;11(5):e0156295. doi: 10.1371/journal.pone.0156295 (PMC4881986; doi:10.1371/journal.pone.0156295)
Supplement: S1 File — Presents a brief overview of the results obtained from each ANOVA and the regression analysis of residuals. (PDF) [file pone.0156295.s009.pdf]

S1 File. Statistical results and tables.

Variability assessment determined that deviations of the logarithm of specific signal remained larger, while those for the background and non-specific binding were commensurate with one another. The ANOVA results (Table B) showed that only the type of measurement impacts the variability. It was found that wg# and power also affected variability. AIC modeling determined that all of the variables were significant, but the residuals for the uncertainty of the specific binding were significantly larger, even after LPSc, wg#, and power were accounted for. Regression analysis (Table C) showed no significance for any of the analyzed variables (LPSc, wg#, and power), but showed that the uncertainty of the specific binding was greater than four times that of the uncertainty in other types of measurements with a p-value = $8.77e^{-16}$. This indicates that the uncertainty in specific binding is significant, but not dependent upon any of the analyzed variables except the inherent variability of LPS itself.

Table A. ANOVA of variable significance (5%) in relation to logarithm of integrated intensity

	DF	Sum Sq	Mean Sq	F Value	Pr(>F)
measurement type	2	62.804	31.402	278.865	<2.2e-16
LPSc	1	1.266	1.266	11.239	0.00109#
wg#	3	1.306	0.435	3.865	0.0113#
power	1	0.52	0.520	4.616	0.0338#
Residuals	112	12.612	0.113		

# indicates numbers with significant p-values for the corresponding coefficient

Table B. ANOVA of Absolute Value of Residuals

	DF	Sum Sq	Mean Sq	F Value	Pr(>F)
measurement type	2	2.691	1.346	45.687	3.10E-15#
LPSc	1	0.028	0.028	0.954	0.331
wg#	3	0.113	0.038	1.279	0.285
power	1	0.053	0.053	1.795	0.183
Residuals	112	3.299	0.029		

# indicates numbers with significant p-values for the corresponding coefficient

Table C. Regression Analysis of Residuals for LPS Concentration Detection

Coefficients	Estimate	Std. Error	t – value	Pr (> | t |)
Intercept	-0.06009	0.15355	-0.391	0.6963
LPSc	0.00013	0.00026	0.521	0.6037
wg1*	0.02712	0.05411	0.501	0.6172
wg2*	0.01488	0.08470	0.307	0.7595
wg3*	0.10956	0.05350	2.048	0.0429#
power	0.00070	0.00052	1.34	0.183
mNSB	0.00378	0.04291	0.088	0.93
mSP	0.40259	0.04291	9.383	8.77E-16#
Residual standard error:		0.17160	on 112 degrees of freedom	
Multiple R-squared:		0.4666		
Adjusted R-squared:		0.4332		
F-statistic:		13.99	on 7 and 112 DF	
p-value:		6.21E-13		

*wg# is a unique identifying number for an individual waveguide
# indicates numbers with significant p-values for the corresponding coefficient
